# Training on contrast-enhanced ultrasound LI-RADS classification for resident radiologists: a retrospective comparison of performance after training

**DOI:** 10.1186/s13244-024-01786-6

**Published:** 2024-08-14

**Authors:** Ting Dai, Hongjing Zhu, Meng Qiao, Yuxuan Song, Yu Sun, Xia Meng, Zhixia Sun

**Affiliations:** 1 China-Japan Union Hospital of Jilin University, No. 126, Xiantai Avenue, Shier Road District, 130033 Changchun, Jilin China; 2 The First Hospital of Jilin University, No. 71, Xinmin Avenue, Chaoyang District, 130033 Changchun, Jilin China; 3 Changchun Sixth Hospital, No. 3188 Yatai Avenue, Kuancheng District, Changchun, 130033 Jilin China

**Keywords:** Liver, Hepatocellular carcinoma, Ultrasonography, Education, Training

## Abstract

**Objectives:**

To evaluate the effects and benefits of training radiology residents on contrast-enhanced ultrasound (CEUS) according to the Liver Imaging Reporting and Data System (LI-RADS).

**Methods:**

In total, 234 patients at high risk of hepatocellular carcinoma (HCC) who underwent CEUS were enrolled, including 27 lesions in the education set and 207 lesions in the test sets (a–d). Forty-five radiology residents and 4 radiology experts involved in CEUS LI-RADS training individually reviewed the test sets before, immediately after, and 3-months after training. The consistency with kappa values of the description of CEUS features, the classification of focal liver lesions (FLLs), and the diagnostic performance were evaluated.

**Results:**

The level of agreement between the radiology experts and residents improved after training (all *p* < 0.05), while there were no significant differences between the post-training and 3-months post-training results (all *p* > 0.05). The sensitivity, specificity, positive predictive value, and area under the curve (AUC) based on the CEUS LI-RADS classification of the radiology experts in the diagnosis of HCC were 62.9%, 96.4%, 96.3%, and 0.796, respectively. The diagnostic performance of the radiology residents significantly improved after training (all *p* < 0.05). Misunderstanding of definitions and subjective interpretation of images were the main reasons for disagreement with multiple responses.

**Conclusion:**

Dedicated CEUS LI-RADS training improved the performance of radiology residents in diagnosing FLLs and their agreement with radiology experts on CEUS features. Images and videos to explain typical features of the training were essential to improve agreement between the radiology experts and residents.

**Critical relevance statement:**

Agreement on lesion descriptors between radiology experts and residents can improve with training.

**Key Points:**

- The diagnostic performance of less experienced radiologists for diagnosing HCC could be improved by training.
- Images and videos to explain typical features during training were essential.
- Agreement on lesion descriptors between radiology experts and residents improved after training.

**Graphical Abstract:**

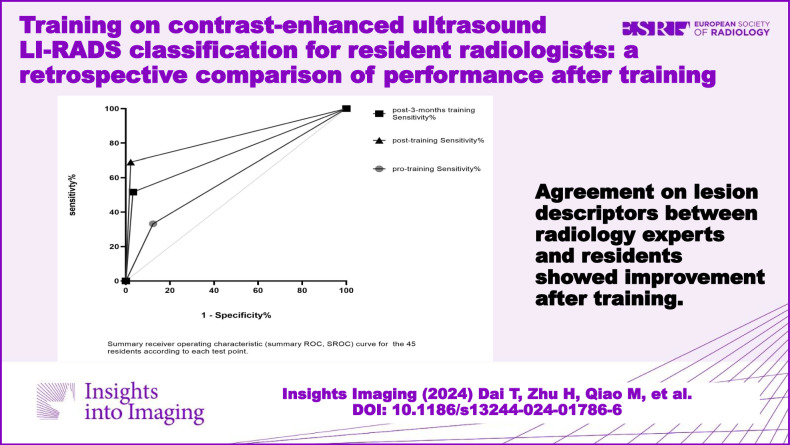

## Introduction

Hepatocellular carcinoma (HCC) is the most common liver malignancy and is associated with an overall 5-year survival rate of less than 12% [1]. Early diagnosis can improve patient prognosis, with a 5-year survival rate of 80% [2]. The majority of HCC cases can be diagnosed based on imaging findings alone [3], and unnecessary biopsies should be avoided. However, for some lesions classified as LR4 (probable HCC) or LR M (probably or definitely malignant but not HCC specific), a biopsy is still needed. Computed tomography (CT) and magnetic resonance imaging (MRI) have been widely used in the diagnosis of focal liver lesions (FLLs) [4], and the diagnostic performance of contrast-enhanced ultrasound (CEUS) is similar to that of CT and MRI [5]. However, compared with CT or MRI, CEUS has greater temporal resolution [6] and can more clearly reflect the arterial enhancement pattern of FLLs. As the contrast agent functions as a pure-blood pooling agent, the washout observed by CEUS is real and clear [7], which is helpful in distinguishing HCC from intrahepatic cholangiocarcinoma (ICC) [8]. The American College of Radiology released a scheme for standardising CEUS reports for FLLs at risk of HCC, which was named the Contrast-Enhanced Ultrasound Liver Imaging Reporting and Data System (CEUS LI-RADS). It is very important for radiologists to perform standardised evaluations of FLLs, effectively communicate with physicians, especially multicentre physicians, and assist physicians in making correct clinical decisions [9].

The European Federation of Societies for Ultrasound in Medicine and Biology (EFSUMB) [10] mandates in its minimum training requirements that CEUS should be performed by experienced observers. In a multicentre international study [11] based on CEUS LI-RADS, the level of agreement between experienced observers reached 0.73, which was better than that between similarly experienced CT/MRI observers; however, the level of agreement between less experienced observers was worse than that between similarly experienced CT/MRI observers [12], especially in evaluating washout. Disagreements in the assessment of FLLs based on CEUS LI-RADS have led to increased false-positive rates, resulting in unnecessary biopsies, excessive radiography, and even excessive treatment, which seriously affect the physical and mental health of patients. Many studies have shown that the level of agreement in lesion evaluation based on the Breast Imaging Reporting and Data System (BI-RADS) and Thyroid Imaging Reporting and Data System (TI-RADS) between less and more experienced observers could be improved by training [13]; however, at present, there is a lack of research on whether training could improve the level of agreement during lesion evaluations based on CEUS LI-RADS between inexperienced and experienced observers.

This study was performed to assess whether training improves diagnostic performance and the level of agreement between inexperienced and experienced observers and to provide specific educational recommendations for observers who need training. Furthermore, we included FLLs with pathological outcomes to assess the diagnostic performance of observers with varying levels of expertise using CEUS LI-RADS, and further evaluated the effectiveness of training.

## Materials and methods

This retrospective study was approved by the ethics committee of our research centre.

### Patients

This study included 234 FLLs from 234 patients who underwent ultrasound and CEUS at our facility between January 2019 and June 2020. For patients with multiple liver lesions, the most suspicious lesion was selected for study. The inclusion criteria were as follows: (1) cirrhosis or chronic hepatitis; (2) detection of malignant lesions and most benign lesions by ultrasound with pathological biopsy results and imaging follow-up for more than 12 months for other benign lesions; and (3) an interval of less than 4 weeks between CEUS and clinical diagnosis. The exclusion criteria were as follows: (1) cirrhosis caused by congenital liver fibrosis or vascular disease; (2) administration of local or systemic therapy; and (3) poor video image quality (except for one patient in the education set). All included lesions met the CEUS LI-RADS criteria.

### CEUS examination

All CEUS examinations in the study were performed according to EFSUMB guidelines [9]. The videos included in the study were acquired by two expert radiologists. The ultrasound contrast agent (25 mg, SonoVue) was mixed with 5 mL of 0.9% saline solution, and 2.4 mL of this suspension was injected through the antecubital vein.

All videos comprised images from both ultrasound and CEUS screens. Videos were recorded continuously from the arrival of microbubbles through the first 60 s; thereafter, images were captured intermittently (every 30 s) to minimise microbubble destruction until the microbubbles had cleared completely from the circulation (4–6 min). The phase of angiographic perfusion was based on CEUS LI-RADS.

### Image selection and interpretation

CEUS videos of 234 liver lesions were selected and reviewed by two expert radiologists dedicated to CEUS examinations based on CEUS LI-RADS and then reviewed by another four expert radiologists. First, four expert radiologists performed the evaluations separately, and then agreement was reached after discussion. The clinical, histopathological, and CT/MRI findings of all videos were not known by the four expert radiologists during the review to minimise bias in ultrasound interpretation. The videos were classified into four groups (a–d), as follows: (a) The education set comprised 27 videos that showed typical CEUS features or were representative of cases described in the CEUS LI-RADS classification. The education set included ultrasound images of the lesions (including the size of the lesions), dual ultrasound and CEUS videos, the consensus results of the classification and CEUS features of the lesions reached by four expert radiologists based on CEUS LI-RADS, and the pathology and CT/MRI findings. The above contents were recorded on a table. The CEUS features included arterial-phase enhancement features, washout, onset of washout, and degree of washout. (2) The three test sets (b, c, d), each containing 69 videos, were organised like the education set. The three tables (b, c, d) could be reviewed by the trainees after the test. The three test sets (b, c, d) were used for the pretraining, post-training, and 3-months post-training evaluations, respectively.

From August 2020 to June 2022, 45 resident radiologists from 9 institutions (2 academic centres and 7 community hospitals) participated in the training. Before training, 45 resident radiologists reviewed test set a. According to the CEUS LI-RADS online, the resident radiologists filled out tables with CEUS features and categories for each lesion. Then, each resident radiologist received theoretical training. Twenty-one resident radiologists participated in the online training, and 24 resident radiologists participated in the offline training. Each training session consisted of three lectures, each of which lasted more than 2 h. The content of the training mainly included the explanation and case presentation of the CEUS LI-RADS. During the training period, 45 resident radiologists were free to review the education set, and 4 expert radiologists answered questions regarding the relevant contents of CEUS LI-RADS during the months of training. Test sets c and d were reviewed, and tables were constructed with CEUS features and categories for each lesion by resident radiologists immediately after training and 3-months after training. The reasons for errors were ascertained with a questionnaire after the three tests. The questionnaire included the following questions: (1) Why did you misclassify the lesion? Please write what you think were the reasons for the classification. (2) If it was a lesion imaging feature recognition error, please state the feature you think, and analyse the reason for the error. To simulate daily diagnosis and prevent “background bias,” the lesions in the three test sets were randomly assigned [14].

### Statistical analysis

All the statistical analyses were performed with SPSS software (version 27) and GraphPad Prism (version 9). Continuous variables are presented as averages ± standard deviations, and categorical variables are presented as percentages. One-way ANOVA and the *χ*^2^ test were used for comparisons between groups. Cohen’s kappa was used to analyse the consistency of the CEUS feature descriptions and categories between the resident and expert radiologists in the three tests, with kappa values ranging from 0.00 to 0.20 indicating slight agreement; 0.20 to 0.40 indicating fair agreement; 0.40 to 0.60 indicating moderate agreement; 0.60 to 0.80 indicating substantial agreement; and 0.80 to 1.00 indicating almost perfect agreement. Multiple responses were used to analyse the reasons for disagreements between the resident and expert radiologists. For the diagnosis of HCC, LI-RADS category 5 results were considered positive, and other results were considered negative. The diagnostic performance of the residents and experienced radiologists was calculated, including the sensitivity, specificity, Positive predictive value (PPV), and accuracy. The area under the receiver operating characteristic (ROC) curve (AUC) was calculated. All *p-*values < 0.05 were considered to indicate statistical significance.

## Results

### Patient and lesion characteristics

**Fig. 1:**
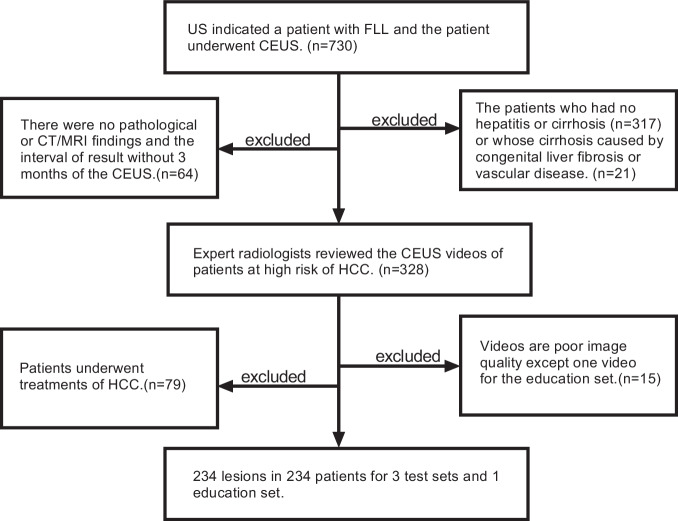
Flow diagram of the study. FLL, focal liver lesion; HCC, hepatocellular carcinoma

A total of 234 patients were enrolled; they included 153 men and 81 women aged between 24 and 83 years (58.3 ± 12.8 years). The exclusion flow chart is shown in Fig. 1. Patient and tumor characteristics are shown in Table 1.

**Table 1:** Patient and lesion characteristics

Variable	Patients (*n* = 234)
Male sex, *n* (%)	153 (65.4)
Age (years), median ± standard deviation (range)	58 ± 12.8 (24–83)
Etiology of hepatitis or cirrhosis, *n* (%)	
HBV	223 (95.3)
HCV	6 (2.6)
Autoimmune hepatitis	1 (0.4)
Alcoholic cirrhosis	1 (0.4)
Non-alcoholic cirrhosis	3 (1.3)
Nodule size (cm), median (range)	3.8 (0.6–14.7)
Histology, *n* (%)	
Hepatapostema	19 (8.1)
Haemangioma	18 (7.7)
Focal fatty change	8 (3.4)
Angioleiomyolipoma	1 (0.4)
FNH	1 (0.4)
RN	20 (8.5)
LGDN	6 (2.6)
HGDN	3 (1.3)
HCC	132 (56.4)
ICC	10 (4.3)
HCC-CC	1 (0.4)
Metastasis	15 (6.4)

Data are presented as the number of lesions, with percentages in parentheses.

*HBV* hepatitis B virus, *HCV* hepatitis C virus, *RN* regenerative nodule, *LGDN* low-grade dysplastic nodule, *HGDN* high-grade dysplastic nodule, *FNH* focal nodular hyperplasia, *HCC* hepatocellular carcinoma, *ICC* intrahepatic cholangiocarcinoma

### Agreement on classifications and CEUS features between resident and expert radiologists

The level of agreement on the CEUS features and classifications of the three test sets between the resident and expert radiologists was evaluated, as shown in Table 2.

**Table 2:** Kappa values of agreement between resident and expert radiologists in assessing FLLs using CEUS LI-RADS

	Pretraining	Post-training	*p*-value^a^	Three months post-training	*p*-value^a^	*p*-value^b^
Rim APHE	0.18 (0.16–0.21)	0.80 (0.73–0.87)	< 0.0001	0.77 (0.71–0.83)	< 0.0001	0.76
APHE	0.63 (0.60–0.66)	0.86 (0.82–0.89)	< 0.0001	0.85 (0.82–0.88)	< 0.0001	0.94
Washout appearance	0.40 (0.36–0.44)	0.84 (0.79–0.88)	< 0.0001	0.83 (0.80–0.87)	< 0.0001	0.99
Late or early washout	0.40 (0.37–0.43)	0.82 (0.77–0.87)	< 0.0001	0.83 (0.80–0.87)	< 0.0001	0.92
Mild or marked washout	0.37 (0.34–0.39)	0.82 (0.77–0.86)	< 0.0001	0.80 (0.75–0.83)	< 0.0001	0.54
category	0.28 (0.25–0.32)	0.78 (0.73–0.84)	< 0.0001	0.78 (0.73–0.82)	< 0.0001	0.97

Data are presented as means, with 95% confidence intervals (CIs) in parentheses

*FLL* focal liver lesion, *CEUS LI-RADS* Contrast-enhanced Ultrasound Liver Imaging Reporting and Data System, *APHE* arterial-phase hyperenhancement

^a^ *p*-values compared to pretraining data

^b^ *p*-values compared to post-training data

Kappa values of 0.81–1.0, 0.61–0.80, 0.41–0.60, 0.21–0.40, and 0.00–0.20 correspond to almost perfect, substantial, moderate, fair, and slight agreements, respectively

Agreement between the resident and expert radiologists was significantly improved post-training and 3-months post-training for CEUS features and classifications (all *p* < 0.05). In the post-training and 3-months post-training evaluations, most resident and expert radiologists showed almost a perfect level of agreement in terms of CEUS features and classifications, except for rim enhancement.

**Fig. 2:**
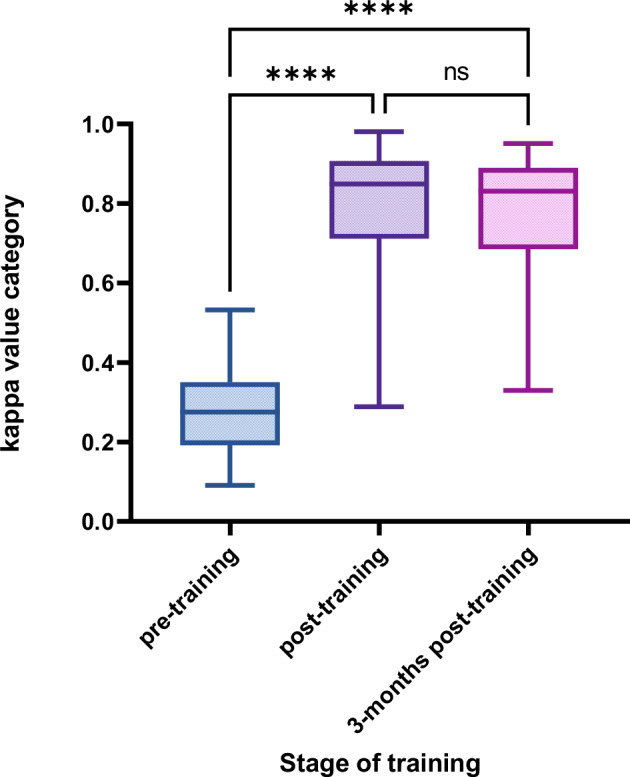
The difference in FLL classification agreement between resident and expert radiologists in the three test sets. FLL, focal liver lesion

Kappa values did not show significant differences in the level of agreement for CEUS features or classifications between post-training and 3-months post-training (all *p* > 0.05). The difference in the level of agreement on FLL classification between the resident and expert radiologists in the three test sets is shown in Fig. 2.

### Diagnostic performance of the resident and expert radiologists

**Fig. 3:**
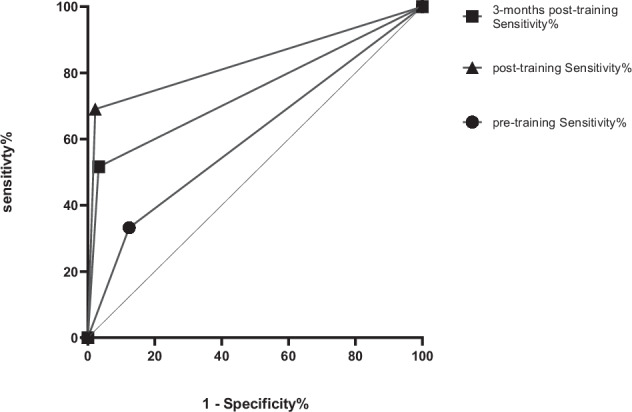
Summary receiver operating characteristic (summary ROC, SROC) curve for the 45 residents according to each test

Compared with the pathological and CT/MRI findings, the accuracy, sensitivity, specificity, PPV and AUC of LI-RADS 5 in the diagnosis of HCC were 76.3%, 62.9%, 96.4%, 96.3%, and 0.796, respectively. The classifications of the expert radiologists and comparisons with the pathology and CT/MRI findings are shown in Table 3. The accuracy, sensitivity, specificity, PPV, and AUC of the diagnosis of HCC by the resident radiologists post-training (75.8%, 69.2%, 97.8%, 99.0%, 0.834) and 3-months post-training (74.5%, 51.6%, 96.8%, 92.0%, 0.742) were greater than those of the resident radiologists pretraining (58.5%, 33.2%, 87.6%, 76.2%, 0.604) (*p* < 0.05), but there were not significant differences in the values between post-training and 3-months post-training (*p* > 0.05, Fig. 3).

**Table 3:** Rates of different cellular types of nodules according to LI-RADS classification by expert radiologists

Lesions *n* (%)	LR-1	LR-2	LR-3	LR-4	LR-5	LR-M
Hepatapostema	18 (7.7%)	0	0	0	0	1 (0.4%)
Haemangioma	14 (6.0%)	0	0	3 (1.3%)	1 (0.4%)	0
Focal fatty change	2 (0.9%)	4 (1.7%)	2 (0.9%)	0	0	0
Angioleiomyolipoma	0	0	0	1 (0.4%)	0	0
RN	0	2 (0.9%)	13 (5.6%)	5 (2.1%)	0	0
LGDN	0	0	3 (1.3%)	3 (1.3%)	0	0
HGDN	0	0	2 (0.9%)	1 (0.4%)	0	0
FNH	0	0	0	0	1 (0.4%)	0
HCC	0	0	8 (3.4%)	8 (3.4%)	84 (35.9%)	31 (13.2%)
ICC	1 (0.4%)	0	0	0	0	9 (3.8%)
HCC-CC	0	0	0	0	0	1 (0.4%)
Metastasis	0	0	0	0	0	15 (6.4%)

Data are presented as the number of lesions with percentages in parentheses

One HCC lesion was classified as uncategorised (LR-NC) in the education set

*RN* regenerative nodule, *LGDN* low-grade dysplastic nodule, *HGDN* high-grade dysplastic nodule, *FNH* focal nodular hyperplasia, *HCC* hepatocellular carcinoma, *ICC* intrahepatic cholangiocarcinoma

### Reasons for disagreements between resident and expert radiologists on CEUS features before training

**Fig. 4:**
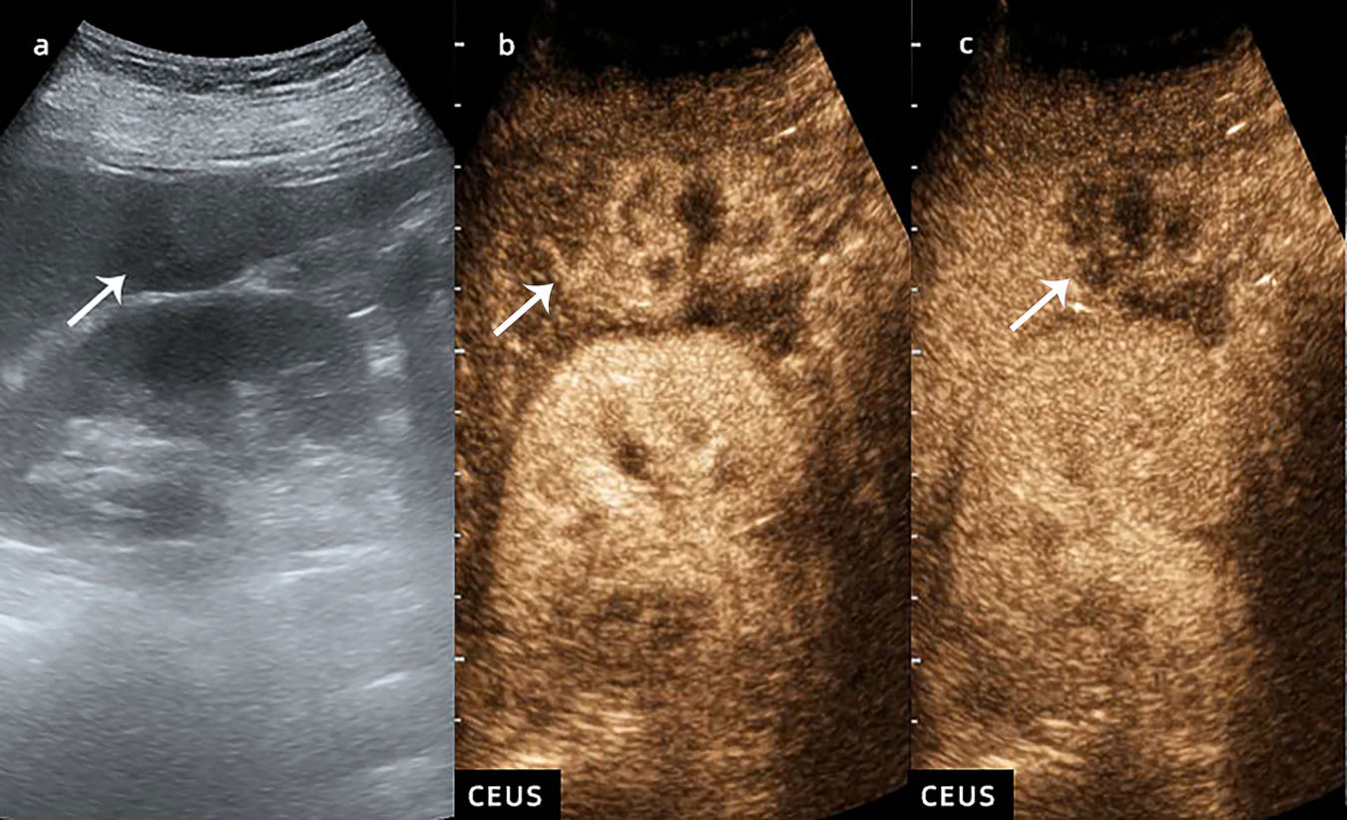
CEUS LI-RADS M (hepatocellular carcinoma). Nodule in a 47-year-old man with hepatitis virus B-related cirrhosis. US image (**a**) showed a hypoechoic lesion sized 4.0 cm in segment VI (arrow). CEUS image (**b**) showed partial APHE during the arterial phase (arrow). CEUS image (**c**) showed early washout during the portal venous phase (arrow)

**Fig. 5:**
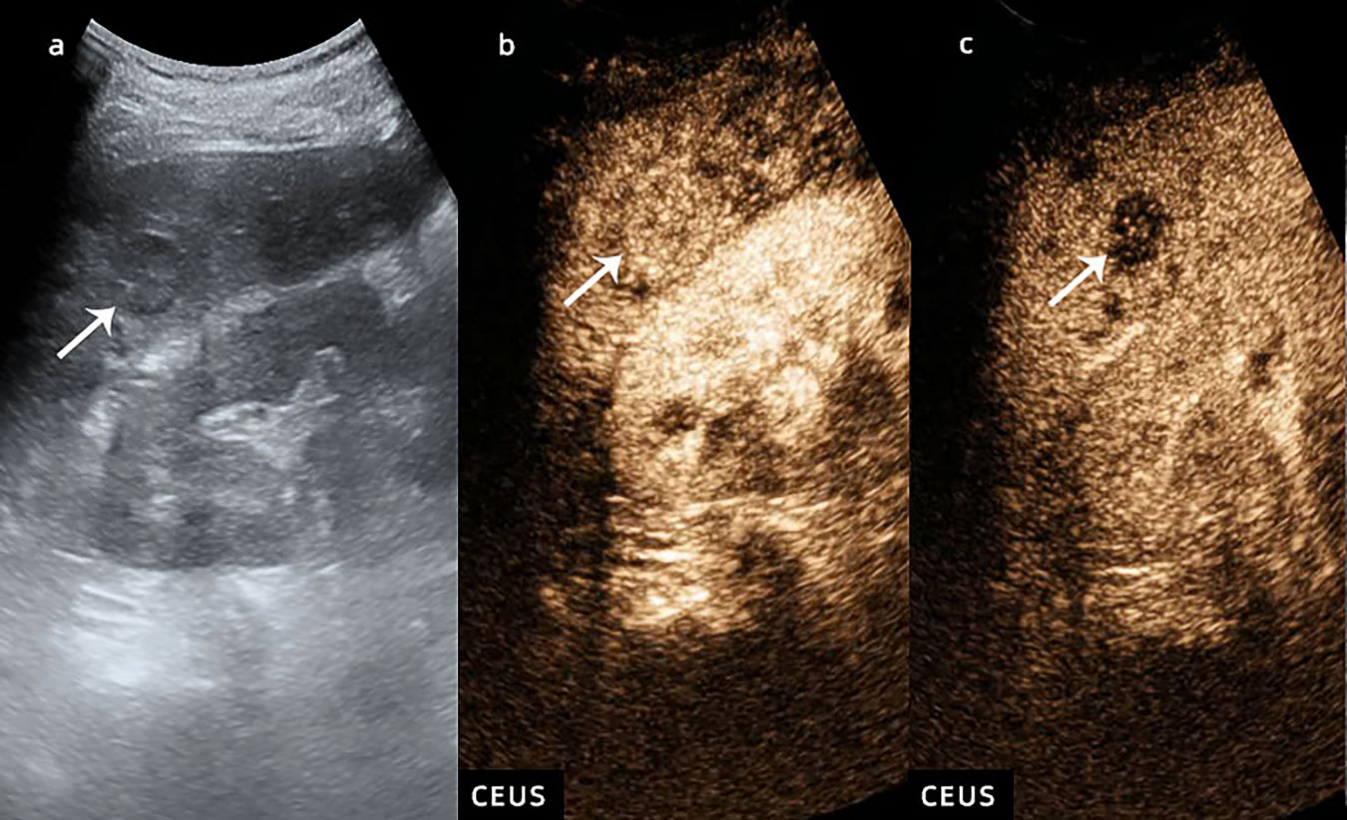
CEUS LI-RADS M (adenocarcinoma of intestinal origin). Nodule in a 68-year-old man with hepatitis virus B-related cirrhosis. US image (**a**) showed a hypoechoic lesion sized 2.1 cm in segment VI (arrow). CEUS image (**b**) showed APHE during the arterial phase (arrow). CEUS image (**c**) showed a marked washout during the late phase (arrow)

A total of 32.6% of disagreements involving arterial-phase hyperenhancement (APHE) were due to subjective image interpretation errors, in which rim enhancement was confused with APHE. A total of 21.5% of disagreements regarding APHE were due to misunderstanding definitions; APHE could not be recognised when multiple enhancement patterns existed (Fig. 4). A total of 93.1% of disagreements regarding rim enhancement were due to image subjective interpretation errors, in which rim enhancement was confused with APHE and discontinuous spherical hyperenhancement. A total of 67.4% of disagreements regarding washout were due to a misunderstanding of definitions; resident radiologists believed that the degree of lesion enhancement in the portal phase was weaker than that in the arterial phase, which could be defined as a washout and partial washout was defined as no washout. A total of 25.8% of disagreements regarding the degree of washout were due to subjective interpretation errors because of differences in subjective descriptions of washout marked as “punched out” and “black” in the guidelines (Fig. 5).

## Discussion

A standardised report based on CEUS LI-RADS is an effective tool for promoting correct clinical decisions in patients at risk for HCC. [15, 16] showed that practitioners should be trained to achieve greater consistency. Our study showed that training could effectively improve the ability of resident radiologists to recognise CEUS features and diagnose HCC. In addition, the resident radiologists showed the same excellent ability to perform CEUS feature recognition and FLL classification in the test 3-months after training, indicating that training can significantly enhance the diagnostic capabilities of resident radiologists over the long term and that the training process is reproducible.

Our study showed that resident radiologists had moderate consistency with expert radiologists in evaluating APHE before training. There was fair and slight consistency between the resident and expert radiologists in terms of evaluating rim enhancement as well as the appearance, onset, and degree of washout and final classification, which are similar to the results of previous studies [17]. Due to the above incorrect recognition of the CEUS features of FLLs, resident radiologists have incorrectly classified FLLs and have failed to effectively diagnose them.

In view of the problems in lesion characteristic evaluation and classification by resident radiologists before training, expert radiologists implemented systematic and targeted norms in the subsequent training. First, the definitions and interpretations of the characteristics and classifications were clarified. APHE could be considered present if the APHE was shown in the entire nodule or only part of the nodule. Washout was defined as a reduction in the enhancement of part or all of the lesion with respect to the surrounding normal liver parenchyma during or after the arterial phase, even if there was no high enhancement during the arterial phase. Second, video-derived images were used to objectify some subjective descriptions. Through the comparison of rim enhancement and discontinuous spherical hyperenhancement, the characteristics of rim enhancement were defined. The image of marked washout was repeatedly clarified by replaying the marked washout videos conforming to the punched out and black features in the education set and in test set a. Finally, timely feedback based on clinical and pathological results after the test could effectively improve ultrasonic interpretation skills [18].

The specificity and PPV of the expert radiologists for HCC diagnosis were 96.4% and 96.3%, respectively, which were similar to the results reported by Terzi, once again confirming the effectiveness of CEUS LI-RADS for diagnosing HCC. In our study, no expert radiologists classified the ICC as LR-5, suggesting a lower risk of diagnosing ICC as HCC based on CEUS LI-RADS. In this study, one patient with focal nodular hyperplasia (FNH) with a background of fatty liver was classified as having LR-5 by an expert radiologist due to APHE and mild washout in the late phase. In addition, another case of haemangioma with APHE and mild washout in the late phase was classified as LR-5. When the haemangioma was accompanied by a shunt in the arteriovenous fistula, there was a mild washout, showing characteristics of malignancy [19, 20]. In the absence of other images and clinical results, expert radiologists classified the lesions as LR-5. CEUS LI-RADS 2017 requires a further standardised description of the diversity of FLLs.

The 45 resident radiologists came from 9 medical institutions of different levels, including 2 community hospitals and 2 academic centres. The diversity of trained personnel is conducive to further research on the possibility of the popularisation of CEUS LI-RADS in medical institutions of different levels for multicentre and multidisciplinary communication. The main limitation of our study was that the trainees played the video on the computer screen. Although the high-quality CEUS videos we selected were sufficient for effectively evaluating the lesions, other differences may have been caused by technical reasons.

Our study suggested that standardised scientific training could improve the performance of resident radiologists in evaluating FLLs, which is conducive to the early diagnosis of HCC. Moreover, images and videos to explain typical features were essential for improving the level of agreement between the radiology experts and residents.

## Data Availability

The data and materials used in this study are available. All data and materials are preserved by the corresponding author and can be obtained by contacting the corresponding author.

## Abbreviations

AUC: Area under the curve
CEUS: Contrast-enhanced ultrasound
CT: Computed tomography
EFSUMB: European Federation of Societies for Ultrasound in Medicine and Biology
FLL: Focal liver lesion
FNH: Focal nodular hyperplasia
HCC: Hepatocellular carcinoma
ICC: Intrahepatic cholangiocarcinoma
LI-RADS: Liver Imaging Reporting and Data System
MRI: Magnetic resonance imaging
PPV: Positive predictive value
